# Food insecurity and dementia risk in US older adults: results from the Panel Study of Income Dynamics

**DOI:** 10.1002/alz.087813

**Published:** 2025-01-09

**Authors:** Cindy W Leung, Noura E Insolera, Julia A Wolfson, Claire T McEvoy, Lindsay H Ryan, Esther M Friedman, Kenneth M Langa, Steven G Heeringa, Wei Hao

**Affiliations:** ^1^ Harvard T.H. Chan School of Public Health, Boston, MA USA; ^2^ University of Michigan, Ann Arbor, MI USA; ^3^ Johns Hopkins Bloomberg School of Public Health, Baltimore, MD USA; ^4^ Centre for Public Health, Queen’s University Belfast, Belfast United Kingdom

## Abstract

**Background:**

Growing research suggests that food insecurity is associated with worse cognitive functioning; however, longitudinal studies are needed to examine food insecurity and dementia risk.

**Methods:**

Using data from the 2013‐2021 Panel Study of Income Dynamics, the longest running nationally representative household panel survey, we examined the effects of food insecurity on dementia risk among 3,232 adults (≥65 years). Food insecurity was assessed biennially using the US Household Food Security Survey Module since 2015. Dementia risk was assessed bienially using the Eight Item Interview to Differentiate Aging and Dementia (AD8) since 2017. We used inverse probability weighting and marginal structural models to account for the time‐varying nature of food insecurity and sociodemographic and health confounders.

**Results:**

In the sample of US households with older adults (mean age (SE), 69.7 (0.2) years, 57% female), the weighted prevalence of food insecurity ranged from 5.3‐7.0% and the weighted prevalence of dementia risk ranged from 20.7‐21.8%. After accounting for baseline and time‐varying confounders, there was a two‐fold association between food insecurity and dementia risk (OR 2.11, 95% CI 1.12, 3.98). In sensitivity analyses, results were similar when expanding the exposure definition to include milder forms of food insecurity, and the outcome definition to include informant‐reported memory loss. Associations were not further modified by sex, race and ethnicity, or household participation in the Supplemental Nutrition Assistance Program.

**Conclusions:**

Food insecurity is a modifiable social determinant of health. Interventions and policies are needed to reduce food insecurity and promote healthy aging for older adults

